# Laparoscopic cholecystectomy for acute cholecystitis in a patient with left-sided gallbladder: a case report

**DOI:** 10.1186/s40792-019-0614-9

**Published:** 2019-04-05

**Authors:** Ryosuke Hirohata, Tomoyuki Abe, Hironobu Amano, Tsuyoshi Kobayashi, Masahiro Nakahara, Hideki Ohdan, Toshio Noriyuki

**Affiliations:** 1 Department of Surgery, Onomichi General Hospital, 1-10-23, Onomichi, Hiroshima, 722-8508 Japan; 2 Department of Gastroenterological and Transplant Surgery, Graduate School of Biomedical and Health Sciences, Hiroshima University, Hiroshima, Japan

**Keywords:** Left-sided gallbladder, Laparoscopic cholecystectomy, Acute cholecystitis

## Abstract

**Background:**

Left-sided gallbladder is a relatively rare anatomical variation that is frequently associated with a biliary system anomaly. Here, we describe a case of left-sided gallbladder with acute cholecystitis treated by laparoscopic cholecystectomy.

**Case presentation:**

An 86-year-old man with acute upper abdominal pain was admitted to our hospital. Computed tomography demonstrated that the gallbladder was centrally dislocated and the wall enhancement was discontinued. Magnetic resonance cholangiopancreatography showed that the gallbladder wall was thickened and abnormally swollen. A laparoscopic cholecystectomy was performed. The round ligament was attached to the right side of the gallbladder, and the left-sided gallbladder was diagnosed by intraoperative findings. The patient was discharged 5 days after surgery without postoperative complications.

**Conclusions:**

A flexible and optimal port site should be inserted in cases of left-sided gallbladder with acute cholecystitis. An assessment of the extra- and intrahepatic biliary system is essential to avoid biliary injury in cases of left-sided gallbladder with acute cholecystitis.

## Background

Left-sided gallbladder (LSG) is defined as a gallbladder whose floor is located on the left side of the round ligament of the liver [1]. Laparoscopic cholecystectomy (LC) is the current gold standard treatment for acute cholecystitis. The 2018 Tokyo guidelines demonstrated that early cholecystectomy under strict criteria could lead to good surgical outcomes [2]. Our previous data showed that early LC within 7 days after symptom onset is a feasible and safe treatment option [3]. It is important to evaluate the preoperative biliary system to prevent intraoperative biliary injury. Of note, the intraoperative biliary injury rate in cases of LSG was around 7% [4], which is extremely high. It is well known that portal and biliary system variations are strongly associated with LSG. Detecting preoperative LSG is difficult due to its rarity and the similarity of its radiological findings to those of gallbladder torsion including gallbladder dislocation and cystic duct twisting [5]. Here, we present a case of acute cholecystitis with LSG following LC with a literature review.

## Case presentation

**Fig. 1:**
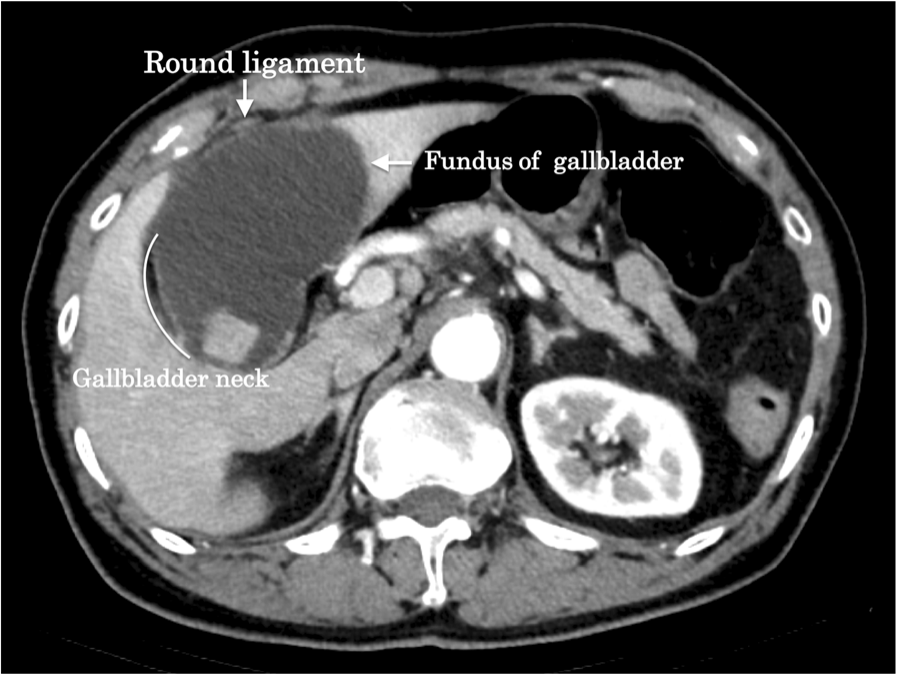
Abdominal contrast-enhanced computed tomography findings. The fundus of the gallbladder was located centrally beyond the round ligament (white arrow)

**Fig. 2:**
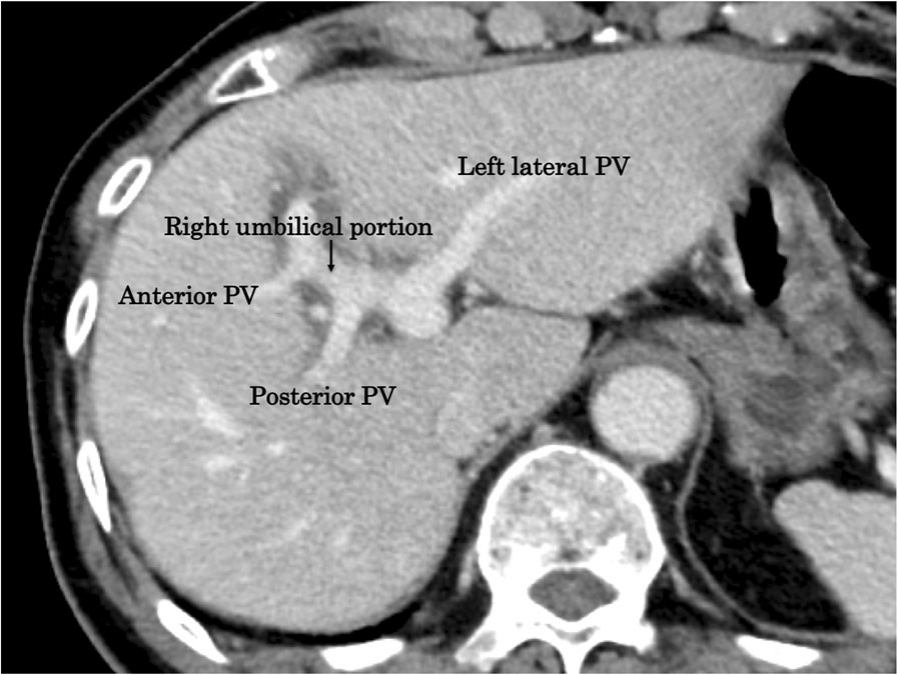
Abdominal contrast-enhanced computed tomography findings. The intrahepatic portal vein formed the right umbilical portion (black arrow)

**Fig. 3:**
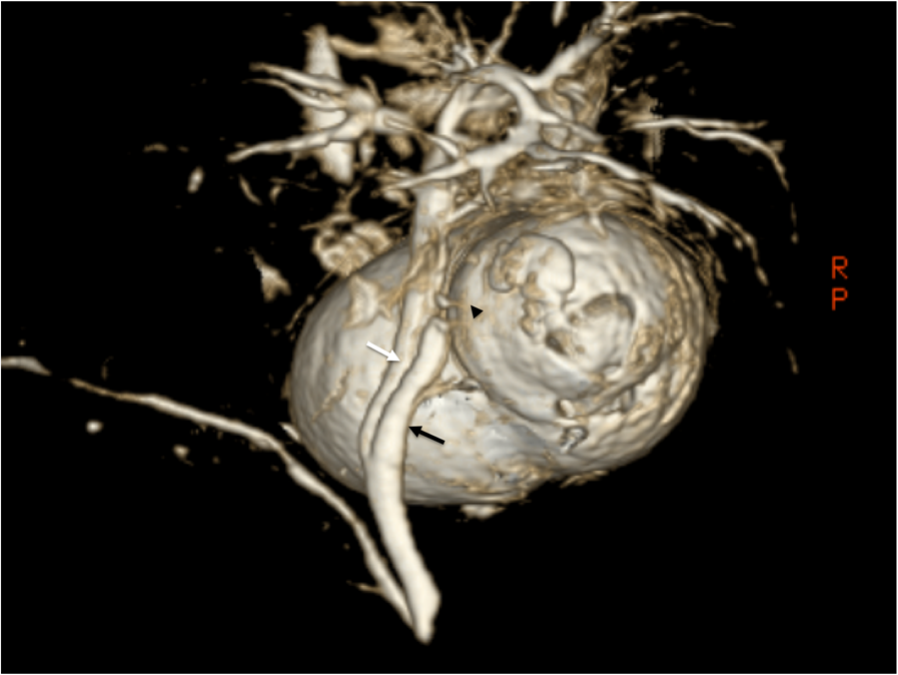
Magnetic resonance cholangiopancreatography findings. The cystic duct (black arrow) branched from the common bile duct (white arrow). The cystic duct was unclear in the middle (black arrowhead)

An 86-year-old man was admitted to our hospital with a 3-day history of acute abdominal pain. The patient had no previous medical history. A physical examination revealed marked right upper quadrant pain with normal bowel sounds. Murphy’s sign was positive. His vital signs were within the normal range. Abdominal ultrasonography revealed an enlarged gallbladder with surrounding fatty tissue inflammation. The blood biochemistry was essentially normal, including C-reactive protein (1.9 mg/dL) and total bilirubin (1.4 mg/dL) levels. An enhanced computed tomography examination revealed an enlarged gallbladder and incarcerated gallstone. Gallbladder wall enhancement was discontinued, and the fundus of the gallbladder was located centrally beyond the round ligament (Fig. 1). The round ligament was attached to the right umbilical portion, which was associated with the anomaly of the intrahepatic portal vein system (Fig. 2). Magnetic resonance cholangiopancreatography demonstrated the root of the cystic duct, while the middle portion of the cystic duct was unclear (Fig. 3).

**Fig. 4.**
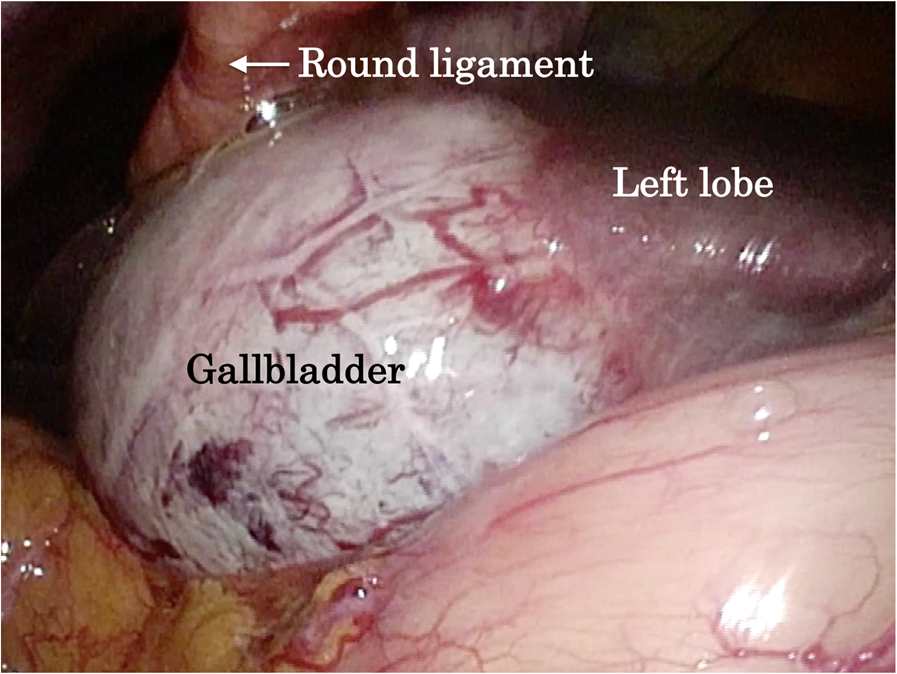
Intraoperative findings. The gallbladder is attached to the liver at the left side of the round ligament

With the preoperative diagnosis of grade II acute gangrenous cholecystitis according to the 2018 Tokyo guidelines or gallbladder torsion, LC was planned. The first port was inserted into the umbilicus, and an enlarged and reddish gallbladder was observed. The gallbladder was swollen; however, torsion was not detected intraoperatively. The second port was placed in the epigastric area, while others were at the right hypochondriac and right lumbar regions. The gallbladder was attached to the left side of the hepatic round ligament (Fig. 4). The cystic duct and the cystic artery were located in the normal positions. Severe inflammation and the narrow working space between the epigastric port and the gallbladder made it difficult to dissect Calot’s triangle; however, the cystic duct and the cystic artery were resected after the critical view of safety was confirmed. Due to the severe inflammation, a subtotal cholecystectomy was finally performed. The operative time was 178 min, and intraoperative blood loss was 50 mL. The pathological diagnosis was acute cholecystitis with a mucosal ulcer. The patient was discharged on the fifth day after surgery without postoperative complications.

## Conclusions

LSG is defined as a rare right-sided round ligament associated with intrahepatic biliary and portal system variations [1]. The incidence of LSG without situs inversus is reportedly 0.2% [6]. Several reports showed that intrahepatic portal vein and biliary system anomalies are rather common in cases of LSG [1, 7]. Attention should be paid to the intrahepatic portal and biliary system as well as cystic duct variations. In our case, the portal vein formed the right umbilical portion, to which the round ligament was attached.

From the hypothesis of LSG occurrence, two types of cystic duct anomalies have been reported [1]: (1) a gallbladder that migrated to the left lobe, that is, a normal cystic duct location, and (2) a gallbladder that formed directly from the left hepatic duct and the cystic duct joining the common bile duct of the left hepatic duct from the left side [1]. In our case, the cystic duct was recognized at the right side of the common bile duct, corresponding to the gallbladder migration type.

The preoperative diagnostic difficulty is due to its rarity and similar radiological findings to those of gallbladder torsion. Gallbladder torsion is highly associated with the floating gallbladder. Gross classified gallbladder torsion into two types: type I is associated with local attachment between the gallbladder and the liver, while type II is complete floating gallbladder [1]. Type I gallbladder torsion can present radiological findings such as central dislocation of the gallbladder fundus and expansion and tapering of the cystic duct on the right side of the common bile duct, which are similar to the radiological findings of our case.

Our previous report demonstrated that the preoperative detection of LSG is important for avoiding biliary injury in cases of hepatectomy [8]. In our case, the anomaly of the intrahepatic portal system was detected preoperatively, but the diagnosis of LSG was made according to intraoperative findings. In cases of acute cholecystitis, it is important to pay attention to the formation of a right umbilical portion as well as the preoperative location of a round ligament to distinguish between LSG and gallbladder torsion.

Patients with acute cholecystitis are generally good candidates for LC as recommended by the 2018 Tokyo guidelines under strict conditions [2]. Several reports have shown the efficacy of early versus delayed cholecystectomy due to the shorter hospital stays, fewer postoperative complications, and higher LC completion rate [2, 3]. Avoiding biliary duct injury (BDI) is a cause for concern when performing cholecystectomy, and it is important to detect an ectopic biliary system in the emergent setting. Clearly detecting Calot’s triangle is essential for avoiding BDI. Regarding the LSG hypothesis, the cystic duct frequently branches from the left hepatic duct, an anomaly that leads to BDI.

Nastos et al. reviewed 18 previous surgical cases of LSG and reported that it can be safely managed by devising of the port arrangement and the use of intraoperative cholangiography [9]. In the literature, the use of an additional retracting port [10] and the fundus first approach [11] have been described. In our case, the distance between the round ligament and the epigastric port was so short that they interfered with each other. It is important to create an adequate workspace between the round ligament and the gallbladder for safe processing of Calot’s triangle. Some reports have stated that manipulation of the falciform ligament is useful [10]. Lifting the falciform and the round ligaments to the body wall using a surgical suture may be an easy approach and enable surgery with a clearer view. In addition, positioning the port more caudally or using an additional port to attract the round ligament would be useful for avoiding obstruction by the round ligament. Here, we performed a conventional American-style LC and attracted the round ligament using the epigastric port. Adopting additional ports or using energy devices may be helpful for ensuring surgical safety and shortening operative time.

In conclusion, LSG is associated with an intrahepatic biliary system anomaly. Here LC was successfully performed. Radiological findings are similar to gallbladder torsion, but identification of the round ligament is useful for the diagnosis of LSG. When LSG is diagnosed, the optimal port site should be determined to ensure safe cholecystectomy.

## Abbreviations

BDI: Biliary duct injury
LC: Laparoscopic cholecystectomy
LSG: Left-sided gallbladder

## References

[1] Gross RE (1936). Congenital anomalies of the gallbladder: a review of one hundred and forty-eight cases, with report of a double gallbladder. Arch Surg..

[2] Takada T (2018). Tokyo Guidelines 2018: updated Tokyo guidelines for the management of acute cholangitis/acute cholecystitis. J Hepatobiliary Pancreat Sci..

[3] Takemoto YK, Abe T, Amano H, Hanada K, Fujikuni N, Yoshida M (2017). Propensity score-matching analysis of the efficacy of late cholecystectomy for acute cholecystitis. Am J Surg..

[4] Abongwa HK, De Simone B, Alberici L, Iaria M, Perrone G, Tarasconi A (2017). Implications of left-sided gallbladder in the emergency setting: retrospective review and top tips for safe laparoscopic cholecystectomy. Surg Laparosc Endosc Percutan Tech..

[5] Bekki T, Abe T, Amano H, Fujikuni N, Okuda H, Sasada T (2017). Complete torsion of gallbladder following laparoscopic cholecystectomy: a case study. Int J Surg Case Rep..

[6] Nagai M, Kubota K, Kawasaki S, Takayama T, Bandai Y, Makuuchi M (1997). Are left-sided gallbladders really located on the left side?. Ann Surg..

[7] Ishii H, Noguchi A, Onishi M, Takao K, Maruyama T, Taiyoh H (2015). True left-sided gallbladder with variations of bile duct and cholecystic vein. World J Gastroenterol..

[8] Abe T, Kajiyama K, Harimoto N (2012). Resection of metastatic liver cancer in a patient with a left-sided gallbladder and intrahepatic portal vein and bile duct anomalies. A case report. Int J Surg Case Rep..

[9] Nastos C, Vezakis A, Papaconstantinou I, Theodosopoulos T, Koutoulidis V, Polymeneas G (2014). Methods of safe laparoscopic cholecystectomy for left-sided (sinistroposition) gallbladder: a report of two cases and a review of safe techniques. Int J Surg Case Rep..

[10] Wong LS, Rusby J, Ismail T (2001). Left-sided gall bladder: a diagnostic and surgical challenge. ANZ J Surg..

[11] Matsumura N, Tokumura H, Yasumoto A, Sasaki H, Yamasaki M, Musya H (2009). Laparoscopic cholecystectomy and common bile duct exploration for cholecystocholedocholithiasis with a left-sided gallbladder: report of a case. Surg Today..

